# Sex identification in rainbow trout using genomic information and machine learning

**DOI:** 10.1186/s12711-024-00944-0

**Published:** 2024-12-30

**Authors:** Andrei A. Kudinov, Antti Kause

**Affiliations:** Natural Resources Institute Finland, 31600 Jokioinen, Finland

## Abstract

Sex identification in farmed fish is important for the management of fish stocks and breeding programs, but identification based on visual characteristics is typically difficult or impossible in juvenile or premature fish. The amount of genomic data obtained from farmed fish is rapidly growing with the implementation of genomic selection in aquaculture. In comparison to mammals and birds, ray-finned fishes exhibit a greater diversity of sex determination systems, with an absence of conserved genomic regions. A group of genomic markers located on a standard genotyping array has been reported to potentially be linked with sex determination in rainbow trout. However, the set of markers suitable for sex identification may vary between populations. Sex identification from genomic data is usually performed using probabilistic methods, where suitable markers are known beforehand. In our study, we demonstrated the use of the Extreme Gradient Boosting approach from the supervised machine learning gradient boost framework to predict sex from unimputed genomic data, when the suitability of the markers was unknown a priori. The accuracy of the method was assessed using four simulated datasets with different genotyping error rates and one real dataset from the Finnish Rainbow Trout Breeding Program. The method showed high prediction quality on both simulated and real datasets. For simulated datasets with low (5%) and high (50%) genotyping error rates, the accuracies were 1.0 and 0.60, respectively. In the real data, the method achieved a prediction accuracy of 98%, which is suitable for routine use.

**Supplementary Information:**

The online version contains supplementary material available at 10.1186/s12711-024-00944-0.

## Background

Information about sex in farm animals is important from both farming and breeding perspectives. At the farm level, the sex of young animals is used to make proper handling actions and management decisions. In a breeding program, sex is used in genetic evaluation, selection decisions, and breeding schemes. For instance, sex is considered a fixed effect in mixed-model equations for genetic prediction or can be used as a criterion for culling animals from a breeding program.

While in some farm species (cattle, pigs, sheep), primary sexual characteristics are easily distinguishable even at a young age, in other species (poultry, fish, insects), the difference between sexes is more visible in adults. Farmed salmonids such as rainbow trout present extreme cases, where sex can be visually identified only when fish start maturing (gonads, coloring, male jaw), which typically corresponds to 2 to 3 years of age [1]. In grown fish without visible sex signs, determining sex requires ultrasound or postslaughter examination, both of which are time-consuming, costly, and sometimes impossible.

Genomic selection [2] has become a popular breeding tool for different species, including farmed fish [3]. For that purpose, massive genotyping of individuals is performed by genome-wide single nucleotide polymorphism (SNP) arrays with a density ranging from 4 to 60K markers. Sex identification in mammals and birds using genomic data is a relatively easy task because both taxa have highly conserved heterogametic genetic sex determination systems, such as sex chromosomes. As a result, it is possible to identify sex by checking the allele type of the conservatively located SNPs in the known parts of a genome. However, there is a high diversity of sex determination systems for ray-finned fishes. Different sex-determining genes interact with environmental effects and epigenetic mechanisms [4, 5]. Therefore, it is difficult to use constant SNPs for sex determination. In salmonid species, including rainbow trout, a male heterogametic sex determination system (sdY region) has been identified [6]. A commonly used genotyping array (Axiom Trout Genotyping Array™) was presented for rainbow trout in 2015, and it includes 41 SNPs marked by USDA that are potentially linked with sex determination [7]. In the array, 15 out of 41 SNPs showed a significant bias towards heterozygosity in males and located in the sdY region [7]. Probabilistic inference methods such as Bayesian approaches [8] are usually used to identify sex from a set of SNPs [9]. This is because not all sex-related markers are expressed similarly in different populations [10] and a certain level of genotyping errors are present. The Bayesian framework implies iterative estimation of the probability that a fish is female over multiple SNPs [8]. In the first iteration, the prior used is 0.5; in the next iterations, the posterior from the previous round is used as a prior. The order in which observations are processed is crucial in iterative Bayesian analysis due to the sequential nature of the updating process. If observations with more reliable data are processed earlier in the sequence, they can have a greater influence on shaping the posterior distribution.

Currently, supervised machine learning approaches are extremely popular across disciplines, including animal breeding [11, 12]. Classification methods such as decision trees, gradient boosting, and others are known to be efficient in binary data prediction. In comparison to the Bayesian approach, machine learning approaches are less sensitive to data quality and not sensitive to the order of the features. This is achieved by multiple data sampling during the model training process. Machine learning approaches have high theoretical potential for use in routine sex identification.

In this study, we demonstrate the possibility to use the extreme gradient boosting (XGB) approach for sex identification in rainbow trout using 15 sex-related SNPs reported by Palti et al. [7]. The demonstrated method is intuitive and can be used in other fish species. Assessment of the method was performed using simulated data, as well as real data from the Finnish National Rainbow Trout Breeding program.

## Methods

### Data

#### Simulated data

Four datasets were simulated. Each dataset included 14,010 fish (5604 males and 8406 females) with 15 SNPs genotyped. The ratio of males to females was set 40:60 to simulate a real Finnish breeding program. The sex of a fish in the data were coded as 0 for males and 1 for females to represent the binary nature of the data. The ground truth model assumption was that males were heterozygous (genotype code 1) and that females were homozygous (genotype code 2) for all 15 markers. For that reason, SNPs were coded as 1 for heterozygotes and 2 for homozygotes. The missing call rate was set to 5% (700 of out 14,010 fish samples per marker) for all markers and all datasets. The samples with missing SNP information were selected randomly. The missing SNP genotypes were coded as 5 and later during an analysis converted to ‘not-a-number’ (nan) instances of NumPy [13].

The four simulated datasets were generated to have different percentages of genotyping errors per SNP. Genotyping error was a deviation from the ground truth model assumption, meaning that the true males and females expressed the wrong genotype. For each SNP, the fish samples with erroneous genotypes were selected randomly from non-missing samples. The percentage of erroneous genotypes (error rate) for a given SNP in the data was calculated as $\frac{{SNP}_{Error}}{{SNP}_{Total}-{SNP}_{Missing}} \times 100$, where *SNP*_*Error*_ is the number of erroneous genotypes, *SNP*_*Total*_ is the total number of genotypes, and *SNP*_*Missing*_ is the number of missing genotypes. The simulated datasets were: (1) *Sim_5,* with a unified error rate of 5% across all SNPs; (2) *Sim_50,* with a unified error rate of 50% across all SNPs; (3) *Sim_rand,* with a unique randomly selected error rate for each SNP in the range from 5 to 50%; and (4) *Sim_real,* mimicking the real dataset with five markers with a randomly selected error rate for each SNP in the range from 5 to 10% and 10 markers with randomly selected error rate for each SNP in the range from 10 to 50%. The error rates are based on a discrete distribution. (Table 1).

**Table 1:** Percentage of individuals for each marker that do not follow the assumption that genotype is homozygous in females and heterozygous in males (error rate) by dataset

Marker	Sim 5^a,b^	Sim 50	Sim rand	Sim real	Real data
AX-89953234	5	50	23	9	51
AX-89955231	5	50	16	6	27
AX-89970231	5	50	36	5	44
AX-89928458	5	50	17	7	47
AX-89941119	5	50	9	8	5
AX-89958463	5	50	26	28	30
AX-89968299	5	50	27	24	9
AX-89924652	5	50	21	54	18
AX-89960682	5	50	22	27	17
AX-89936452	5	50	33	39	48
AX-89947083	5	50	8	25	54
AX-89955288	5	50	13	38	38
AX-89950690	5	50	44	32	53
AX-89926028	5	50	29	37	28
AX-89963605	5	50	14	55	47

^a^Error rate computed as SNP_Error_/(SNP_Total_ − SNP_Missing_) * 100, where SNP_Error_ is the number of erroneous SNP samples, SNP_Total_ is the total number of SNP samples, and SNP_Missing_ is the number of missing SNP samples. The average proportions of SNP_Missing_ were 5% and 6% in the simulated and real data, respectively

^b^Sim 5 = simulated data with a 5% missing rate for all SNPs; Sim 50 = simulated data with a 50% missing rate for all SNPs; Sim rand = simulated data with a random error rate in the range of 5 to 50%; Sim real = simulated data with a random error rate in the range of 5 to 10% for 5 SNPs and 10 to 50% for 10 SNPs; real data = data from the Finnish Rainbow Trout Breeding Program

#### Real data

Genomic and phenotypic sex information were obtained for 1362 fish (491 males and 871 females) reared at the nucleus of the Finnish Rainbow trout breeding program [14, 15]. For the present study, a subset of fish born between 2014 and 2019 was used, as genomic and phenotypic data were available only for individuals born in 2014, 2018, and 2019. Pedigree was available only up to 2019. The fish were genotyped using the Axiom Trout Genotyping Array™ array. From unimputed and unfiltered genotypes, 15 sex-related SNPs [7] were extracted. The markers were coded the same as in the simulated data. It was assumed that there was no difference between alternate homozygotes, as the male genotype has a bias toward heterozygosity [7]. Missing alleles were converted to ‘not-a-number’ (nan) instances. No imputation of genomic data was performed. The observed average missing call rate across the 15 SNPs was 2.8%. Table 1 presents percentage of samples per SNP which phenotypically were males but genomically homozygous and phenotypically females but genomically heterozygous.

The phenotypic sex of the fish was coded as 0 for males and 1 for females. For fish born in 2014, phenotypic sex was determined based on the pedigree (i.e., if an individual was used as a sire or a dam in the matings). For fish born in 2018 and 2019, sex was recorded based on visual signs (coloring, male jaw) and ultrasound examination at 2 and 3 years of age. The proportion of males to females in the breeding program matings was 40:60.

## Prediction method and validation

### Gradient boosting

The Extreme Gradient Boosting (XGB) approach from the supervised machine learning gradient boost framework was used for solving the classification problem [16]. The algorithm is an ensemble learning method that implies parallel iterative ($k$) training of gradient boosted decision trees: ${f}_{k}\left({\varvec{x}}\right)$, where ${\varvec{x}}$ is the matrix of SNP genotypes. The use of a binary logistic loss term ensures that the predicted probabilities align with the true labels (known sex) and can be expressed as $\sum_{i=1}^{n}\left[{y}_{i}*log\left(1+\exp \left(-{\widehat{y}}_{i}\right)\right)+\left(1- y_{i} \right)*log\left(1+\exp \left({\widehat{y}}_{i}\right)\right)\right] + \sum_{k=1}^{K}\Omega ({f}_{k}),$ where $n$ = number of training samples, ${y}_{i}$ = recorded sex for the fish *i* (0 or 1), ${\widehat{y}}_{i}$ = predicted log-odds of sex, $K$ = number of trees in the ensemble, and $\Omega ({f}_{k})$ = regularization term for the $k$-th tree. The regularization term was computed as ${\gamma T}_{k}+\frac{1}{2}\lambda {\sum }_{j=1}^{{T}_{k}}{\omega }_{jk}^{2}$, where ${T}_{k}$ = the number of leaf nodes for the $k$-th tree, $\gamma$ = the number of leaf nodes, ${\omega }_{jk}$ = the weight of the j-th leaf node in the $k$-th tree, and λ = parameter that controls the squared leaf node weights. The model hyperparameters that were used during model training were (i) ‘learning rate’—scaling parameter for each tree, (ii) ‘individual tree depth’—maximum tree depth allowed, (iii) ‘features sampling’—number of SNPs randomly sampled for each tree, and (iv) ‘data sampling’—portion of the data randomly sampled for each tree. The best model hyperparameters were systematically selected and fine-tuned for each dataset separately, using the GridSearchCV algorithm implemented under python scikit-learn package v.1.4.2 [17]. The purpose of this algorithm is to find the best possible combination of parameters using a grid search and a five-fold cross-validation procedure. The process evaluates the performance of each parameter combination across multiple partitions of the data on the test and training datasets, ultimately identifying the configuration with the highest average accuracy. The grid of parameters used in GridSearchCV algorithm are in the supplemental materials (see Additional file 1: Text S1). There was no restriction on the number of samples required to create a node during the tree construction process (i.e., minimal size parameter was set to 0). The XGB model was implemented based on the xgboost python package [18].

### Validation procedure

Five- and twofold cross validation approaches were used to evaluate the accuracy of sex prediction using the XGB model, in which the data was randomly split into five or two equal folds, respectively. In the fivefold cross-validation, each fold iteratively acted as a test group, and the remaining folds as a single training group. The numbers of samples in the training and test groups were 2802 and 11,208 in simulated and 272 and 1090 in real datasets. In each iteration, a training group was used to train the XGB model. Thereafter, the trained model was used to predict sex in the testing group by using only the SNP genotypes. The accuracy of prediction was calculated in the testing group using the predicted and known sex as ${{N_{Correct} } \mathord{\left/ {\vphantom {{N_{Correct} } {N_{Total} }}} \right. \kern-0pt} {N_{Total} }}$, where ${N}_{Correct}$ is the number of correct predictions and ${N}_{Total}$ is the total number of samples in a test. The twofold cross-validation approach was similar to the five-fold cross-validation approach, but only one training-test iteration was used. The number of samples in the training and testing groups were equal (7005 and 681). This approach presents the most extreme case of validation, where the training set is equal to the test set. For each dataset, five- and twofold cross-validation was repeated 20 times, and the average accuracy was reported.

Relative marker importance was computed using the ‘feature_importances’ algorithm of XGB [16]. The algorithm created multiple data splits and measured the reduction in the loss-function on a single SNP of every split—gains. The gains from a single SNP were summed and compared with the gains of other SNPs to determine the importance of SNPs. The SNP importance values were normalized to one hundred. Higher relative marker importance implies better prediction quality when corresponding marker presented.

## Results and discussion

The average accuracies of sex prediction using different datasets are shown in Table 2. Differences between the average accuracy in five- and twofold cross-validation approaches in different datasets were small (from 0.01 to 0.02). The XGB model was robust to cases where a larger part of the data was masked. The *Sim_5* and *Sim_50* simulation scenarios were used to present extremes of possible genotyping error rates, and a prediction accuracy differed substantially between them; none of the samples were misclassified in the *Sim_5* scenario, while in the *Sim_50* scenario, approximately 40% of the samples obtained a wrong sex. The accuracies for *Sim_rand* and *Sim_real* were relatively high (0.995 and 0.998, respectively), suggesting successful XGB model training. The accuracy of *Real_data* from the Finnish breeding program (0.979) was lower than that in the *Sim_real* scenario because the data structure was better in *Sim_real* than in *Real_data*. In *Sim_real,* five markers were assumed to have an error rate less than 10%, while in *Real_data,* two markers (AX-89968299 and AX-89941119) had an error rate less than 10%, and two (AX-89960682 and AX-89924652) had an error rate less than 20% (Table 1).

**Table 2:** Average accuracy of sex prediction in the tested datasets

Data set^a^	Five-fold cross validation		Twofold cross validation	
	Accuracy^b^	Number of misclassified samples^c^	Accuracy	Number of misclassified samples^d^
Sim 5	1.000	0	1.000	0
Sim 50	0.600	1120	0.599	2804
Sim rand	0.995	12	0.995	34
Sim real	0.998	12	0.997	19
Real data	0.979	5	0.977	15

^a^Sim 5 = simulated data with a 5% missing rate for all SNPs; Sim 50 = simulated data with a 50% missing rate for all SNPs; Sim rand = simulated data with a random error rate in the range of 5 to 50%; Sim real = simulated data with a random error rate in the range of 5 to 10% for 5 SNPs and 10 to 50% for 10 SNPs; Real data = data from the Finnish Rainbow Trout Breeding Program

^b^Accuracy calculated as N_Correct_/N_Total_, where N_Correct_ is the number of correct predictions and N_Total_ is the total number of predicted samples

^c^Number of test samples: simulated data—2802; real data—272

^d^Number of test samples: simulated data—7005; real data—681

**Fig. 1:**
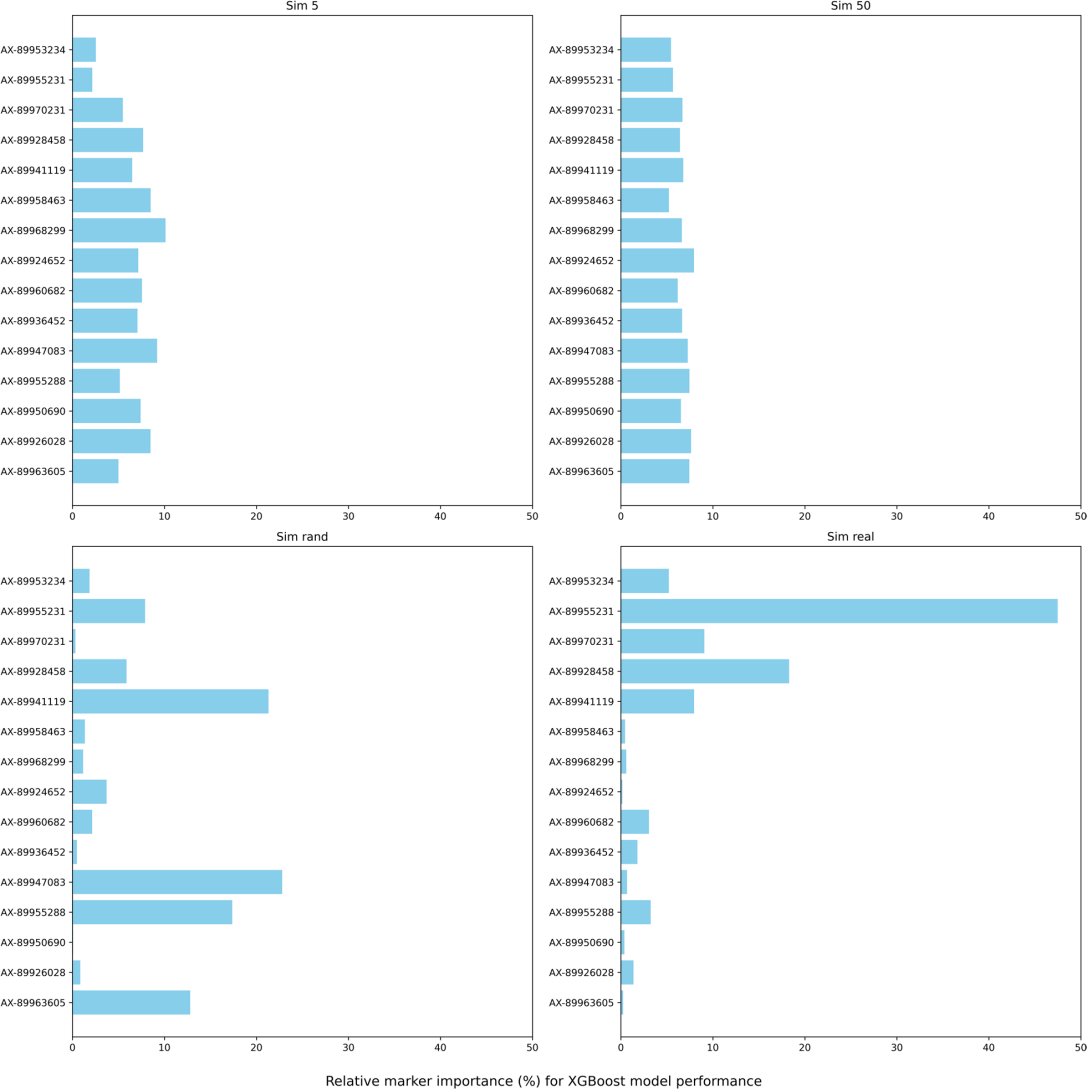
Relative marker importance for successful sex prediction using the XGB model in simulated datasets. The relative marker importance was determined as the sum of gains in prediction quality over data splits. SNPs were compared within the model and normalized to one hundred, with higher values indicating better prediction quality. The sum of relative importance across SNPs is 100%

**Fig. 2:**
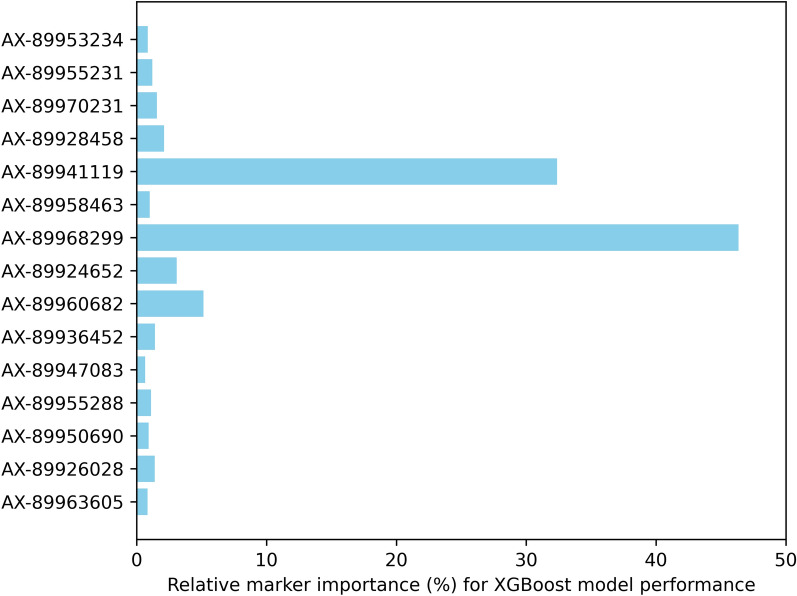
Relative marker importance for successful sex prediction using the XGB model in the Finnish Rainbow trout data. The relative marker importance was determined as the sum of gains in prediction quality over data splits. SNPs were compared within the model and normalized to one hundred, with higher values indicating better prediction quality. The sum of relative importance across SNPs is 100%

The relative marker importance plots (Figs. 1 and 2) reveal as the most important markers with the lowest error rates by the model.

The highest relative importance (> 5%) was assigned to five markers in *Sim_real* and to three markers (AX-89968299, AX-89941119, and AX-89960682) in *Real_data*. Three markers from *Real_data* can be used for targeted genotyping and prediction of sex, but this approach will neglect possible changes in marker expression in a population within years. The prediction error rate in the *Real_data* dataset was considered low (1 to 2%), and the XGB model can be used in the Finnish Rainbow Trout Breeding Program routine.

Choice of model hyperparameters was performed in an automated way to ease the training of the model for application in commercial computing routines. The best hyperparameters for each dataset are presented in Table 3. A complicated data structure requires a larger fraction of the data to be used during the model training process. The values of the parameters ‘subset of features’ and ‘data sample’ were closer to 1 (aka 100%) in *Sim_50*, *Sim_rand*, *Sim_real* and *Real_data*, indicating that a large number of SNPs and fish samples were used during the training process. The parameter ‘individual tree depth’ was shallow (2) in the *Sim_5*, *Sim_50*, and *Sim_real* datasets, for different reasons. In *Sim_5,* all markers were informative; hence, to prevent overfitting, construction of a larger tree was restricted. In contrast, many SNPs were noninformative in the *Sim_50* and *Sim_real* scenarios. As a result, constructing a large tree was not a sufficient approach, instead, XGB used a larger fraction of the data during model training, increasing the chance that most informative markers would be present in each subset of the data. The relative marker importance values were low and similar for all SNPs in *Sim_5* and *Sim_50* (Fig. 1). The parameter ‘data sample’ is key to understanding marker information content. All markers in *Sim_5* were closely related to a small ‘data sample’ (0.05), indicating high information content in every marker (i.e., each marker had good prediction ability). In contrast, ‘data sample’ was large (0.90) in *Sim_50,* meaning that none of the markers had superior prediction ability.

**Table 3:** Best XGB model parameters for each dataset obtained via grid search cross-validation

Data set^a^	Subset of features	Learning rate	Individual tree depth	Data sample
Sim 5	0.01	0.05	2	0.05
Sim 50	0.40	0.10	2	0.90
Sim rand	0.30	0.05	6	0.80
Sim real	0.80	0.20	2	0.10
Real data	0.60	0.30	6	0.90

^a^Sim 5 = simulated data with a 5% missing rate for all SNPs; Sim 50 = simulated data with a 50% missing rate for all SNPs; Sim rand = simulated data with a random error rate in the range of 5 to 50%; Sim real = simulated data with a random error rate in the range of 5 to 10% for 5 SNPs and 10 to 50% for 10 SNPs; real data = data from the Finnish Rainbow Trout Breeding Program

The association of SNPs with sex is known to be dynamic in various populations of the same species [1]. The Bayesian iterative approach proposed for sex prediction [8] may suffer from so-called sequential analysis error, i.e. it is sensitive to the quality and order of markers used in the iterative solving process. Thus, the resulting posterior distribution (predicted sex) can be significantly biased if noninformative SNPs are presented in the first iteration rounds of an iterative Bayesian analysis. For example, Calboli et al. [9] reported the use of only 7 out of 41 potential markers for sex prediction, as the quality of the 7 markers was high [*Calboli CFC personal communications*]. Accordingly, some marker preselection can be performed before the actual sex determination process. In contrast, the XGB algorithm is robust to the order and presence of noninformative markers in the data, as indicated by the high prediction power in the *Sim_rand* and *Sim_real* datasets. This method is attractive for use in routine breeding programs because it does not require any prior knowledge on the expression of markers in a population and no imputation of genomic data needs to be performed. It is worth noting that XGB performed best on individuals with the same genomic structure as the fish used to train the model. Using a training population that is distant from the testing population may reduce the accuracy of prediction. For example, in our test, prediction of sex in the *Real_data* using XGB model trained on the *Sim_real* data yield 21% of wrong sex predictions.

It is always a good approach to retrain models occasionally using new phenotypic information. An error rate is expected when phenotypic data are collected in a commercial environment. For instance, sex may be incorrectly recorded for fish that do not act as parents due to unclear visual signs or low ultrasound quality. To mitigate this, the prediction model can be improved by weighting the recorded sex according to the source of the phenotypic information, as sex observations recorded on parental status can be considered more reliable than those based on visual signs alone. In addition, Fraslin et al. [1] reported high genomic heritability estimates for spontaneous maleness in XX rainbow trout. Thus, even if all fish in the training set are recorded based on parental status, it is wise to perform control sdY genotyping to validate the accuracy of molecular sex identification in the populations of interest.

The integrated learning algorithm XGB is an easy tool for solving large classification problems. The possibility of efficient handling missing data without imputation, high accuracy of prediction, and robustness to overfitting makes the algorithm suitable for complicated datasets, including genomic data. The XGB models reduce model complexity and support parallel computation to effectively reduce the training time. Other machine learning algorithms, such as support vector machines, random forests, or neural networks, can be used as alternatives. However, support vector machines and neural networks are more complex than XGB models and it is wise to maintain a balance between task and model complexity for lower computational resources and to favor simpler solutions over complicated ones. Although random forests might provide predictions as accurate as the XGB model, with easy-to-interpret results, the XGB model has better on-fly customization options, including the ability to handle missing values and assign weights within the model.

## Conclusions

We demonstrated the use of the XGB Machine learning approach for sex identification in simulated and real rainbow trout genomic data. The proposed method allowed prediction of sex without imputation and the use of both informative and noninformative SNPs. Model robustness was demonstrated by using differently designed simulated datasets. The accuracy of prediction in the data from the Finnish Rainbow trout breeding program was 98% in both two- and five-fold cross-validation, which is suitable for routine use of the method.

## Supplementary Information

Additional file 1: Text S1. Grid of hyperparameters used in GridSearchCV approach.

## Data Availability

The simulated data, scripts for the simulated data and XGB models, and trained XGB models used in the study are available at Github URL: https://github.com/andeeri-k/trout_sex_determination. The marker data from the Finnish Rainbow Trout Breeding Program is not available as it belongs to the National Breeding program.
