# Supplementary material for: Sex identification in rainbow trout using genomic information and machine learning
Source: Genet Sel Evol. 2024 Dec 30;56:79. doi: 10.1186/s12711-024-00944-0 (PMC11687024; doi:10.1186/s12711-024-00944-0)
Supplement: Supplementary file 1 — Additional file 1: Text S1. Grid of hyperparameters used in GridSearchCV approach. [file 12711_2024_944_MOESM1_ESM.docx]

Grid of hyperparameters used in GridSearchCV approach for finding best possible combination of parameters

Subset of features: [0.01, 0.1, 0.2, 0.3, 0.4, 0.5, 0.6, 0.7, 0.8, 0.9, 1.0]

Learning rate: [0.05, 0.1, 0.15, 0.2, 0.25, 0.3]

Individual tree depth: [2, 3, 4, 5, 6, 7, 8, 9, 10, 11, 12, 13, 14, 15]

Data sample: [0.05, 0.1, 0.2, 0.3, 0.4, 0.5, 0.6, 0.7, 0.8, 0.9]
